# A comparative review of ORC and R-ORC technologies in terms of energy, exergy, and economic performance

**DOI:** 10.1016/j.heliyon.2024.e40575

**Published:** 2024-11-20

**Authors:** Serdal Damarseckin, Sebe Yves Junior Kane, Ayhan Atiz, Mehmet Karakilcik, Haci Sogukpinar, Ismail Bozkurt, Saadin Oyucu, Ahmet Aksoz

**Affiliations:** a Sırnak University, Faculty of Engineering, Department of Energy Systems Engineering, 73000, Sırnak, Turkey; b Department of Physics, Faculty of Sciences and Letters, University of Cukurova, Adana, 01250, Turkey; c Department of Electric and Energy, Vocational School, University of Adiyaman, Adiyaman, 02040, Turkey; d Department of Mechanical Engineering, Faculty of Engineering, University of Adiyaman, Adiyaman, 02040, Turkey; e Department of Computer Engineering, Faculty of Engineering, Adıyaman University, 02040, Adıyaman, Turkey; f MOBILERS Team, Sivas Cumhuriyet University, 58350, Sivas, Turkey

**Keywords:** Thermal and exergy efficiencies, Energy conversion, ORC and R-ORC, Electricity generation, Organic fluids

## Abstract

This review examines Organic Rankine Cycle (ORC) technology, which generates electricity using organic fluids at low temperature ranges. To enhance the efficiency of basic ORC systems, they are often adapted into Regenerative Organic Rankine Cycle (R-ORC) systems. The review highlights the dimensions of economic, energy, and exergy efficiency, which are critical for practical application. Factors like the choice of working fluid, heat source temperature, and heat exchanger efficiency significantly affect economic feasibility; suboptimal choices can reduce returns and hinder project viability. Strategic decisions can improve economic outcomes and make ORC technology more appealing, as improved efficiency often leads to better economic performance through increased energy output and reduced operational costs. ORC and R-ORC systems promote sustainable energy production by enhancing energy efficiency in various applications, including geothermal power plants, industrial waste heat recovery, biomass energy production, and solar power plants. By enabling electricity generation even at low temperatures, these systems efficiently utilize existing energy sources, reduce dependence on fossil fuels, and minimize environmental impacts, thus providing both economic and ecological benefits. Additionally, when the studies conducted are examined, R-ORC exhibits higher performance than basic ORC. R-ORC is significantly superior to ORC in terms of both energy and exergy efficiency. Specifically, in terms of energy efficiency, R-ORC has been found to be 1.83 %–25.5 % more efficient. Regarding exergy efficiency, R-ORC demonstrates approximately 7.69 % better performance. Furthermore, due to these increases in efficiency, it has been determined that R-ORC also provides a more positive economic contribution compared to ORC. Thus, comparisons between ORC and R-ORC systems play a significant role in sustainable energy production and offer valuable guidance for future research. The limitations of ORC and R-ORC systems include limited efficiency due to low temperature differentials, the environmental impact of the organic fluids used, and high costs.

## 1 Introduction

The relationship between climate change and energy consumption represents one of the most significant environmental challenges of our modern world. The interaction between these two issues has global-scale environmental, economic, and social consequences. Climate change gives rise to various problems worldwide, including increasing temperatures, rising sea levels, intense weather events, and loss of biodiversity. One of the root causes of these problems is the excessive dependence on energy sources associated with high carbon emissions because of fossil fuels. When energy is derived from carbon-emitting sources such as coal, oil, and natural gas, it results in the emission of greenhouse gases into the atmosphere. This contributes to the ongoing issue of global climate change and environmental degradation. The increasing concentration of these greenhouse gases accelerates global temperature rise, further intensifying the impacts of climate change. To better understand the relationship between climate change and energy consumption, it is essential to examine the role of the energy sector [1].

Determining the energy and exergy efficiency of any system provides crucial information for evaluating it from a thermodynamic perspective. Energy efficiency reflects how effectively a system uses input energy to produce useful outputs, such as work or heat, aiming to maximize output while minimizing energy loss. Exergy efficiency measures a system's performance by considering not only the quantity of energy but also its potential to perform useful work. This indicates whether the energy is fully utilized and reflects the system's capacity to generate valuable work.

Energy production and consumption from fossil sources contribute significantly to greenhouse gas emissions worldwide. Therefore, shifting energy production to cleaner and more sustainable sources is a critical step in the fight against climate change. Clean energy sources, which encompass solar and wind energy, hydroelectric generation, and geothermal energy, present environmentally friendly alternatives that substantially diminish carbon emissions in stark contrast to the use of fossil fuels. These sustainable energy options play a pivotal role in mitigating climate change and lessening the environmental impact. These sources produce little to no carbon emissions in comparison to fossil resources. Additionally, energy efficiency plays a crucial role. Enhancing energy efficiency not only leads to decreased energy consumption but also plays a crucial role in curbing the emission of greenhouse gases. This practice not only conserves valuable resources but also contributes significantly to our efforts to combat climate change and reduce our environmental footprint [2]. Thermal energy is seen an effective tool in efforts to reduce climate change because it helps decrease the use of fossil fuels and promotes the adoption of clean energy sources, thus having a positive impact on environmental sustainability. Thermal energy can assist in reducing greenhouse gas emissions by limiting energy production based on fossil fuels while meeting energy demands [3]. Thermal energy systems, including solar, geothermal, and ocean thermal energy, are notable for their lower or even zero carbon emissions during energy production, effectively mitigating the release of greenhouse gases into the atmosphere. Furthermore, these systems significantly improve energy efficiency, contributing to a more sustainable and efficient utilization of energy resources [4]. The ORC enables the effective conversion of thermal energy, playing a critical role in the process of converting heat obtained from hot sources into electrical energy. The ORC system operates on the principle of producing heat at high, medium and low temperatures and converting this heat into mechanical energy by expanding a fluid operating at low temperatures. This approach serves the effective use of thermal energy resources and focuses particularly on solar energy, geothermal sources and industrial excess thermal energy. In this way, we utilize our energy resources efficiently, access sustainable alternatives and encourage environmentally friendly practices. ORC technology is seen a significant step in decreasing the use of fossil fuels and reducing carbon emissions [5]. R-ORC is a special type of Rankine cycle for converting thermal energy into electricity. This system is important for energy performance and sustainable energy generation. R-ORC converts thermal energy more efficiently, reducing energy losses and increasing electricity production. Additionally, this technology has the potential to convert thermal energy from renewable energy resources such as biomass and solar into electricity. This feature contributes to sustainable energy generation and decreases carbon footprint by reducing dependence on fossil fuels. Such systems offer a more environmentally friendly and economical approach in the energy sector and industrial processes [6].

ORC and R-ORC cycles stand out as advanced cycles that significantly support sustainability in energy conversion, especially at a time when the global emphasis on renewable energy sources is more pronounced than ever. These innovative cycles contribute to the transition toward sustainable energy systems by effectively transforming various thermal energy sources into electricity, thereby enhancing energy accessibility and reliability. They are utilized across a diverse range of applications, including geothermal energy production, optimization of solar thermal systems, and the recovery of waste heat in industrial processes, which are all critical for improving energy efficiency in today's world. By capturing and converting waste heat into useable energy, ORC and R-ORC cycles play a crucial role in advancing sustainable energy production and reshaping the future of the energy sector in an environmentally conscious manner. This study provides a comprehensive examination of the energy and exergy efficiencies, economic aspects, and application areas of ORC and R-ORC technologies, offering insights into their operational effectiveness and viability. Additionally, it conducts a detailed comparative analysis of these two cycles to understand performance differences and evaluate which system performs better under specific operational conditions, thereby aiding stakeholders in making informed decisions. This comparative analysis not only informs decision-making on energy efficiency and economic sustainability but also addresses environmental impacts, revealing each system's unique environmental benefits and potential for increased energy output along with reduced operating costs. By comparing ORC and R-ORC systems, the study contributes to optimizing system designs for various applications, which can significantly enhance the efficiency and effectiveness of renewable energy solutions. Beyond this, the research highlights best practices, identifies areas for improvement, and fosters an environment of innovation and sustainability in energy technologies. In this context, the research serves as a valuable resource for researchers, policymakers, and industry practitioners who are aiming to develop and implement energy systems that meet the demands of an increasingly competitive global market while minimizing environmental impacts. Moreover, since no in-depth study currently exists that comprehensively compares these two technologies in the literature, this research not only fills an existing knowledge gap but also serves as a foundational guide for further studies and projects focused on sustainable energy solutions, ultimately contributing to the advancement of the field and supporting the global transition towards a more sustainable future.

To improve understanding of this study, research on ORC and R-ORC have been categorized into experimental and theoretical studies, which are further divided into single and multiple working fluid studies within each category.

## 2 Energy, exergy and economic assessment of the ORC

**Fig. 1:**
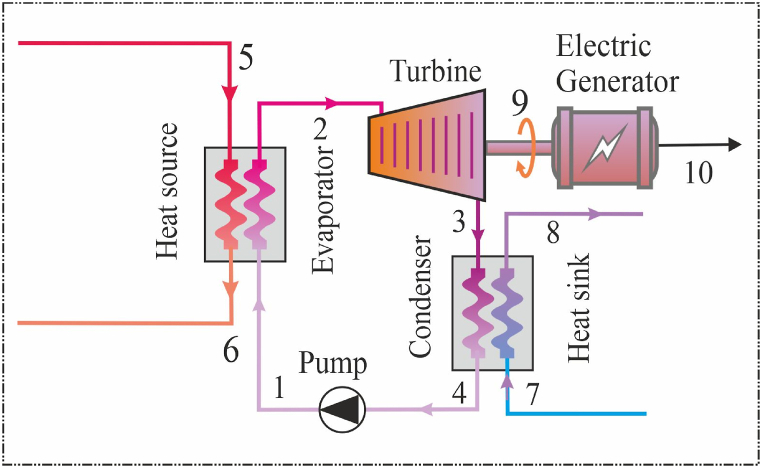
The operating principle of the ORC or B-ORC.

The ORC or BORC (Basic Organic Rankine Cycle) system includes four basic components as shown in Fig. 1. These components work together to facilitate the operation of the system. These four main components are: evaporator, expander, condenser and pump. The functioning of the ORC involves a series of carefully orchestrated steps to harness thermal energy and convert it into mechanical work and, ultimately, electricity [7]. The process begins with the pump, which delivers the working fluid (referred to as state 1) to the evaporator and the selecting fluid is exposed to a heat source in the evaporator, causing it to absorb heat and transition from a liquid to a vapor state. This high-pressure vapor (point 2) is then directed into the expander (turbine). Within the expander, the high enthalpy of the vapor is effectively converted into mechanical work, a critical step in the energy conversion process. As the vapor expands and performs mechanical work, its pressure and temperature decrease, and it exits the expander as low-pressure vapor (point 3). As the turbine rotates, it drives a generator that converts mechanical energy into electrical energy. The vapor at low pressure, indicated as point 3, is then directed into the condenser, where it experiences a transition in its phase. In the condenser, typically with the assistance of water or another cooling medium, the vapor is rapidly cooled and condensed back into a liquid state. Vapor to liquid conversion is a vital step in the ORC process. After the resulting liquid condenses in the condenser, it becomes present at the condenser from point 4. This liquid is then pumped back into the evaporator, completing the cycle and starting a new energy conversion cycle. The ORC system is designed to efficiently capture thermal energy and convert it into mechanical work. Therefore, it stands out as a valuable technology in a wide range of applications, such as the use of thermal energy resources for sustainable and environmentally and friendly electricity generation.

ORC is an extremely important technology for energy conversion and is of great importance for the following reasons [8]:

1. Sustainable Energy Production: ORC has the capacity to convert heat obtained from hot sources (e.g. solar, geothermal or industrial waste thermal energy) into electrical energy. This contributes to a more effective use of renewable and sustainable energy resources.
2. Waste Heat Evaluation: Recovering waste heat generated in industrial facilities or energy production processes increases energy efficiency and contributes to more efficient use of energy resources.
3. Providing Energy in Remote and Isolated Areas: ORC technology can be used to generate electricity in remote areas or isolated areas that do not have access to the electrical grid. This plays an important role in meeting basic energy needs.
4. Carbon Reduction: ORC systems help reduce carbon emissions from fossil fuels, playing an important role in combating climate change.

For these reasons, ORC acts an effective role in the energy sector and sustainability efforts and is considered an effective tool for energy transition. Since ORC is an important technology for the above reasons, many studies have been done on it to find the most efficient and economical situation.

### 2.1 Experimental studies for ORC

Experimental research plays a crucial role in validating theoretical concepts by providing empirical evidence that supports or challenges existing models. It allows researchers to investigate real-world applications, uncover unexpected results, and refine hypotheses based on observed data.

In an experimental study, it was analyzed a small-scale ORC (1 kW capacity) employing R134a as the operating fluid. It utilized low-grade thermal energy resources (75 °C, 85 °C, and 95 °C) with helical evaporators, condensers, and a car compressor. The experiment found that the ORC had an optimum performance of 3.33 % and power output of 279.58 W, based on an input temperature and pressure of 67.7 °C and 1.25 MPa [9]. In addition, renewable resources like solar, geothermal, and excess thermal energy can power the ORC due to its need for working at low-temperature. An experiment utilizing solar radiation as a heat resource with R134a in a small ORC system achieved a maximum efficiency of 4.30 % and 185.9 W output at a 95 °C heat source temperature. In this solar energy system, ORC increases the usability of the system by producing energy even at low temperatures [10]. In another experimental study, multi-objective optimization is used to design competitive products in renewable energy. This study optimizes an ORC system for electric efficiency and heat exchanger area to indirectly minimize plant cost. Four Pareto optimal solutions are identified, with electric efficiency ranging from 14.1 % to 18.9 % and heat exchanger areas from 446 m^2^ to 1079 m^2^, using octamethyltrisiloxane as the working fluid [11]. An additional experimental investigation delves into the evaluation of a combined heat and electricity generation cycle, which seamlessly integrates a gas turbine and an ORC via a thermal energy recovery steam generator. The study reveals that the greatest exergy losses are encountered within the combustion chamber, and it also uncovers that enhancing specific design parameters leads to an enhancement in thermodynamic efficiency while simultaneously escalating the overall capital costs of the system. Raising air preheater temperature benefits the system, while increasing certain temperature differences reduces efficiencies and raises costs [12]. In another experimental study conducted for different organic fluids, a model was created to compare different operating fluids at a low-temperature in ORC. The study compares and optimizes three different ORC configurations using medium-temperature geothermal water, the investigation reveals that the fundamental ORC utilizing R245fa and powered by geothermal water at a temperature of 150 °C stands out as the top performer in both thermodynamic and economic aspects. Additionally, among 14 working fluids, R141b emerges as the top choice based on various evaluation methods for comprehensive examination encompasses exergo-economic analysis, dual-objective optimization, and grey relational analysis as integral components of the study [13]. For example, in an experimental work discusses the use of ORCs in power plants, highlighting their advantages over traditional Rankine cycles. It analyzes 16 organic fluids as working mediums and proposes a methodology for integrating ORCs into cogeneration processes. Dry fluids are preferred for ORCs, enabling regeneration and improving thermal efficiency. The feasibility of integration hinges upon the thermal energy rejection characteristics exhibited by the underlying industrial process. It's shown that appropriate integration can reduce grid power consumption, enhance energy efficiency, and reduce environmental effect. The selecting of operating fluid is crucial, and economic factors should be considered before implementation [14]. Another experimental study, in response to the fossil energy crisis and environmental pollution, a two-stage series ORC with double thermal energy resources is proposed to efficiently utilize heat sources of varying temperatures, improving power generation and economic benefits. Comparative analysis demonstrates that this system has the lowest electricity generation for investment payback time and cost, making it ideal for low-temperature thermal energy resources [15]. Another experimental study employing Non-Dominated Sorting Genetic Algorithm-II (NSGA-II) was conducted on a cogeneration system comprising of a 30 MW gas turbine and an ORC cycle. Key decision variables included compressor ratios, turbine efficiencies, and temperatures. Results showed increased exergy efficiency from 51.4 % to 56.15 %, a reduction in total cost ratio from $5460/h to $4751/h, and a decrease in environmental cost ratio. The exergo-economic parameter improved from 10.68 to 28.503, highlighting the trade-off between efficiency and cost. Gas turbine inlet temperature played a crucial role, while optimizing temperatures in the evaporator and heat recovery steam generator led to improved performance [16]. In another experimental study, an all-encompassing decision-making framework has been introduced, aiming to facilitate the selection of the most suitable operational fluids (R245fa, R600, R1234ze, R1234yf, R236ea, RC318, R134a, R142b, R124, and R22) for excess thermal energy recovery employing an ORC in a container ship. The chosen working fluid, R245fa, was determined based on considerations of thermodynamics, economics, safety, and environmental factors, resulting in an electricity generation cost of $0.0596/kWh, with the Multi-Objective Grey Wolf Algorithm and NSGA-II algorithms demonstrating similar performance in the analysis [17].

### 2.2 Theoretical studies for ORC

The ORC is a widely employed technique for generating electricity by transforming thermal energy into electrical power. This method is commonly used in various applications to harness heat sources and convert them into a valuable source of electrical energy. The ORC works by evaporating higher molecular weight liquids using organic fluids instead of water vapor. In certain studies, a singular organic fluid was employed as the working medium, whereas in other investigations, the performance of the ORC was assessed through the utilization of multiple organic fluids. The theoretical studies provide an important foundation for the design and optimization of ORC systems, helping to support the results obtained with practical applications.

A research theoretical study delves into the analysis of a low-temperature ORC employing R134a as the working fluid. It systematically investigates the impacts of varying temperature and pressure conditions on the performance of the ORC system. The findings of this research indicate that the utilization of R134a in the ORC system proves to be highly suitable for energy generation, highlighting its efficacy in harnessing low-temperature heat resources for electricity generation [18]. For slightly higher temperatures in another theoretical study, the study discusses a thermodynamic analysis on utilizing a low-temperature heat resource (up to 150 °C) in a subcritical ORC with R134a as the operating fluid. The study explores how varying input pressure, temperature, and discharge pressure affect cycle efficiency. Results show that efficiency is not strongly influenced by just increasing temperature but improves with higher pressure ratios and input temperatures. Additionally, the effect of adding an internal heat exchanger is examined, resulting in maximum efficiencies of 11 % and 14 % for the basic ORC and an internal heat exchanger [19]. Another similar theoretical study analyzes the utilization of a solar-assisted the ORC with R245fa as the operating fluid for power generation. This system integrates a solar subsystem with a central receiver and ORC. The research identifies exergy destruction rates in each component, emphasizing the central receiver (52.5 %) and heliostat field (25 %) as major contributors. In addition, the energy efficiency of the system is 9.95 %, while the exergy efficiency is 10.66 % [20]. In another theoretical study, the organic fluids R245fa and R123 were first tested individually and then mixed at ratios ranging from 10 % to 90 % to investigate the best electrical and thermal efficiency of the ORC. As a result of the study, it was observed that using R245fa/R123 at an optimal mixing ratio of 0.6/0.4 increased the system's electricity generation by 12.87 % and improved its efficiency by 4.84 % compared to the 0.7/0.3 ratio. These improvements were observed with increasing operating temperature, while factors such as evaporation pressure and flue gas operating temperature also affected the system's efficiency [21]. In another theoretical study comprehensively evaluates the thermodynamic performance of a zero-emission, solar-powered trigeneration system through energy and exergy analyses. The system produces electricity, heating, and cooling using an ORC and a single-effect H₂O/LiBr absorption heat pump. As a result of optimization, an energy efficiency of 152.4 % and an exergy efficiency of 21.1 % were achieved, with the primary exergy losses attributed to the solar collectors, responsible for 73 % of the exergy destruction. The system's performance is particularly sensitive to variations in the solar field outlet temperature and the ORC condensation temperature; thus, controlling these parameters plays a critical role in regulating energy production [22]. Another theoretical paper discusses the simulation of a small-scale ORC facility using low-temperature excess thermal energy for electricity generation. It employs a steam generator, a scroll compressor modified as an expander, and the R245fa operating fluid. The simulation, conducted using MATLAB and Cool-Prop, yielded a maximum performance of 9.6 % and 17 kW net power. The study explores the impact of component characteristics on system performance and anticipates future experimental validation. Findings suggest that the ORC system can operate more efficiently with increased hot source energy [23]. Another different theoretical work, it found ammonia, R1234yf, and R507a to be suitable for Ocean Thermal Energy (OTE). Additionally, a sensitivity analysis showed that the choice of fluid had minimal impact on the model, with key factors being hot source temperature and heat exchanger characteristics [24]. Another theoretical paper explores power generation from waste heat using an ORC with five different pure working fluids. It emphasizes the importance of avoiding superheating, as it negatively affects thermal efficiency. The ORC configuration with an internal heat exchanger (IHE) and isopentane as the operating fluid achieves the maximum thermal performance of 13.16 %, outperforming other configurations by 1.4 %–1.38 % and highlighting the significance of IHE in improving efficiency and maximizing net power output [25]. A separate theoretical research endeavor focuses on the optimization of ORCs tailored to the efficient recovery of waste heat from a hypothetical aluminum manufacturing facility situated in Norway, featuring the utilization of two distinct high-temperature streams. It evaluates 102 working fluids, finding HFE-347mcc as the best pure fluid (85.28 % exergy efficiency) and isobutene–isopentane as the top mixture (3.3 % power increase). RE347mcc is the best choice for lower and higher electricity prices, while HFO-1336mzz faces challenges due to evaporation heat and regenerator size [26]. Another theoretical study examines ORC design and thermodynamic optimization for low-capacity heat sources. Among the R134a, R290, R32, R152a, R125, R1270, R1234ze, R1234yf fluid groups, R152a and R1234ze were found to perform particularly well than others [27]. Another theoretical study analyzes the performance of an ORC with various fluids, including R134a, R236fa, R245fa, R600a, R717, and R718, based on heat source temperature and heat exchanger effectiveness. Taguchi statistical method evaluates parameter importance, with waste-heat source temperature having the greatest impact (59.80 %) and heat exchanger effectiveness the least (2.18 %). The best and worst operating conditions yield thermal efficiencies of 15.26 % and 8.61 %, respectively [28]. ORC plays a significant role as a thermal energy conversion technology that captures waste heat and converts it into electrical energy by utilizing low-temperature heat sources, aiming to enhance energy efficiency in industrial processes and, in turn, increase economic value by using resources more effectively. This theoretical work analyzes 16 different organic fluids in basic and modified ORCs. Additionally, a methodology for the effective integration and optimization of ORC with a background process is presented, demonstrating the benefits of integration through illustrative examples [29]. A separate theoretical study delves into a thermo-economic assessment involving a multi-objective optimization approach, focusing on the comparative analysis of the performance and cost aspects when employing either pure working fluids or mixtures within ORCs. Four models are considered with different temperature points for mixture working fluids. The impact of mass ratio and various key factors on exergy performance and levelized energy cost is analyzed. The results show that the efficiency of mixtures compared to pure operating fluids depends on various factors, and model 2 is found to be favorable for its high thermodynamic efficiency and lower economic parameter in four model [30].

### 2.3 Experimental and theoretical studies for ORC

Both theoretical and experimental studies play a crucial role in advancing the understanding and development of ORC systems, as theoretical analyses provide foundational insights into the thermodynamic principles and efficiencies, while experimental research validates these theories and reveals practical challenges, ultimately leading to optimized designs and improved performance in real-world applications.

In a separate research endeavor, an experimental and theoretical study was conducted to explore the potential of a pentagonal rotor rotary expander within an ORC, with the aim of efficiently harnessing low-temperature and small-scale excess heat. Compared to a triangular rotor, the pentagonal rotor expander generated 80 % more power. The ORC system with the pentagonal rotor expander achieved 140 W of power generation and a 1.7 % efficiency, showing potential for increased waste heat recovery in this system [31].

## 3 Energy, exergy and economic assessment of the R-ORC

**Fig. 2:**
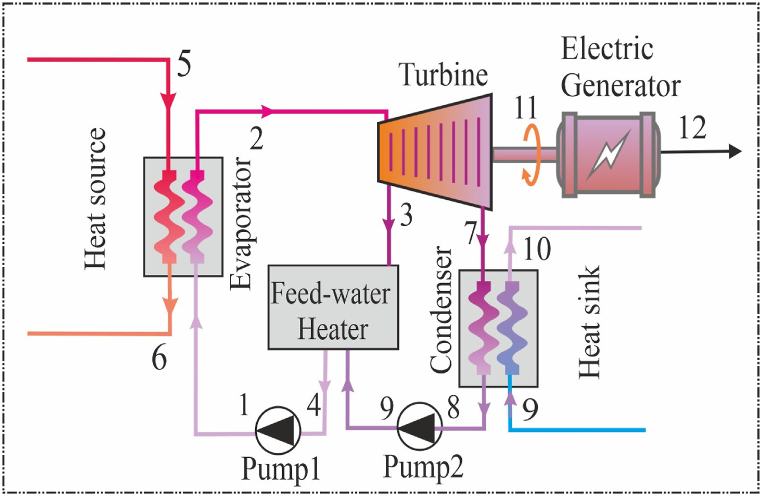
The working principle of the R-ORC.

Fig. 2 shows one of regenerative ORC which begins with a heat source, providing thermal energy to the system, which is then absorbed by an organic working fluid. This fluid vaporizes into high-temperature, high-pressure vapor and enters a high-pressure turbine, generating power and reducing vapor pressure and temperature. In Fig. 2, an essential component in the ORC is the inclusion of a feed-water heater. In this arrangement, the steam withdrawn from the turbine is combined with the feed-water as it exits the pump. The objective is for this mixture to leave the heater in an ideal state, transitioning into a saturated liquid at the heater's operating pressure [32].

The R-ORC stands as a pivotal thermal cycle with significant implications for sustainable energy generation. This cycle is used to generate electrical energy from heat sources, typically low-temperature waste heat. R-ORC is a type of ORC and utilizes organic operating fluids. In this cycle, the heated organic operating fluid vaporizes, expands through a turbine, and then produces electrical energy through a generator. An important feature of R-ORC is the application of thermal energy recovery within the cycle. This enhances the performance of the system as excess heat is reused within the process, allowing us to use resources more efficiently. As a result, industrial facilities, geothermal sources, solar energy plants, and other heat sources can generate more energy. R-ORC is widely used in sustainable energy production with the aim of reducing environmental impacts, improving energetic performance, and lowering the carbon footmark. Additionally, by converting waste heat into a valuable energy source, it helps improve the balance between energy consumption and production. Therefore, R-ORC is recognized as a significant innovation in the energy sector [33].

### 3.1 Experimental studies for R-ORC

An experimental study focuses on enhancing the efficiency of the ORC for geothermal applications. By introducing regenerative preheating in a novel ORC test rig, a 9.9 % increase in thermal performance is carried out, particularly beneficial for combined heat and power (CHP) production. The new ORC-CHP architecture, combined with district heating systems, can lead to a 9.4 % boost in yearly electricity generation and improved economic prospects for geothermal projects [34]. Another experimental investigation assesses an excess thermal energy recovery system utilizing a regenerative ORC with a 2 MW natural gas engine. Advanced exergetic analysis categorizes components into avoidable/unavoidable and endogenous/exogenous groups to identify efficiency improvement opportunities. Pentane emerges as the best operating fluid, with an important impact on net power based on operating conditions. Toluene as an operating fluid in R-ORC yields the best overall performance than pentane, hexane, and octane. Condenser and evaporator exhibit the most exergy destruction and offer the greatest potential for system improvement. Further optimization and computational fluid dynamics analysis are recommended for future studies [35]. In a separate experimental study, the utilization of the zeotropic mixture isopentane/R245fa proved to be more effective, resulting in a remarkable 10.54 % enhancement in power output and a substantial 9.55 % increase in fuel efficiency when applied to a combined diesel engine and R-ORC under the engine's rated operating conditions [36].

### 3.2 Theoretical studies for R-ORC

R-ORC technology is of significant importance, and substantial research has been dedicated to its development and advancement. For example, a theoretical investigation is dedicated to the enhancement of a CO_2_ transcritical Rankine cycle designed for the recuperation of waste heat from internal combustion engines. This innovation involves the integration of a preheater and a regenerator, employing CO_2_-based binary zeotropic mixtures as the working fluids. Upon optimizing the pump speed, the system utilizing the mixtures showcases a notable increase in net power output by 6.34 kW and a 2.19 % enhancement in thermal efficiency when compared to the utilization of pure CO_2_ as the operating fluid [37]. An innovative theoretical solar thermal electric generation system with an R-ORC and small concentration ratio CPCs is discussed. The system increases electrical efficiency by 4.9 % at an irradiance level of 750 W/m^2^; This efficiency increase is achieved primarily due to improved ORC efficiency, although collector efficiency decreases due to higher temperatures [38]. In a theoretical study, a low-temperature flue gas excess heat-driven R-ORC system is examined. The study presents a simplified design approach and identifies crucial temperature thresholds that dictate the necessity of a regenerator and its effect on the efficiency of the ORC [39]. In another theoretical work, it is focused on designing, analyzing, and optimizing an R-ORC using dry working fluids. Parametric calculations were conducted to assess thermodynamic performance for 14 dry working fluids, considering operating conditions and fluid properties. Regression analysis provided thermal efficiency equations for various ORC configurations. The inclusion of regeneration enhanced the uniformity of thermal efficiency among different fluids. The specific net power generation, however, remained constant with the introduction of regeneration. Butane, isobutene, and R113 presented the maximum specific net power generation. Environmental and safety factors influenced working fluid selection. The results revealed logarithmic correlations between thermal efficiency and cycle parameters. The validation of the EPV-11 model was consistent with the findings in the literature [40]. Another theoretical study is dedicated to the choosing of highly dry operating fluids for R-ORC systems, optimizing their extraction conditions for various heat sources. Heptane is the ideal choice for closed heat sources at 0.45 MPa and 478.64 K, whereas pentane, hexane, propylcyclohexane, undecane, and o-xylene are suitable options for various open heat sources with specific conditions [41].

## 4 Comparison of energy, exergy and economic assessments the ORC and R-ORC

Comprehensive comparison of energetic, exergetic and economic analyses of ORC and R-ORC systems reveals the potential advantages of these two thermal conversion technologies in electricity generation from renewable and non-renewable energy resources in terms of sustainability and economic gains of energy conversion and it has a guiding importance in the design and optimization of future energy production systems.

### 4.1 Experimental studies for ORC and R-ORC

Many research works have been conducted to compare these two technologies. For instance, an experimental study investigates whether the addition of a regenerator to ORC systems improves the overall performance of the system by providing additional heat recovery from the exhaust. A 10 kW ORC system was used as an experimental prototype and compared with the ORC and R-ORC versions. In the experiments, the heat input was set in the range of 30–90 kW and R245fa was used as the working fluid. The study defined a “heat source temperature utilization ratio” for the effective utilization of the heat source temperature and thus set a benchmark standard to measure the utilization efficiency of the heat source. Detailed performance analyses of three main cycles in the experimental setup – the heat source cycle, the ORC cycle and the cooling water cycle – were performed and the system behavior was investigated under different scenarios. It was observed that the thermal efficiency of the RORC system could reach 5.5 %, which was approximately 25.5 % higher than the thermal efficiency of ORC, which was 4.1 %. This result reveals that the regenerator provides higher thermal efficiency through heat recovery [42]. In another experimental study conducted for the same organic fluid under different conditions, the study designed an R-ORC using biogas from domestic waste in Belgium, optimizing it for power production and efficiency. R245fa was chosen as the operating fluid. The subcritical R-ORC outperformed the supercritical and simple ORC systems, offering improved performance without safety or investment drawbacks [43]. In another experimental study, two-stage parallel and series ORCs (PTORC and STORC) were presented to upgrade thermal energy improvement performance utilizing the same fluid. STORC exhibited superior performance in reducing irreversible losses and upgrading net electricity generating compared to PTORC, making it the preferred choice for engineering applications [44]. An experimental study compared an ORC with R-ORC employing R123 as the operating fluid. ORC had higher power generation and thermal performance at low evaporating temperatures, making it appropriate for low-temperature thermal energy resources. R-ORC outperformed ORC at high evaporating temperatures, making it preferable for high-temperature heat sources due to its higher thermal efficiency [45]. Another experimental study, it used R123 as the operating fluid to investigate two configurations: ORC and R-ORC. Two scroll expanders, 1# with a 66 mL/r suction volume and 2# with an 86 mL/r suction volume, are examined under same thermal energy resource circumstances. The results show that 2# expander has higher electricity generation and speed but minimum isentropic performance. The best power output occurs at a filling factor of 0.8–0.9 in BORC using 2# expander, achieving a maximum thermal efficiency of 2.96 %. The different expander suction volumes significantly affect system efficiency by altering mass flow rates [46]. Another experimental study focuses on improving the performance of the R-ORC utilizing R123 as the operating fluid for converting low-temperature thermal energy resources into electricity. A specially designed technology was introduced to address R123 leakage issues, and a throttle valve was used to protect the turbine during startup and shutdown. Experimental tests with a geothermal resource at 130 °C demonstrated a 7.98 % efficiency for the R-ORC, which is 1.83 % higher compared to ORC [47]. Another experimental study focuses on optimizing regenerative Clausius and ORC cycles with two feed-water heaters employing EES software. It utilizes artificial neural networks and artificial bee colony algorithms to optimize thermal efficiency, exergy efficiency, and specific work. This approach is applied to both water and R717, providing intriguing insights into power cycle optimization. The results reveal that specific net power output, thermal and exergetic performance initially increase and then decrease with changes in pump outlet pressures. Optimal pump outlet pressures for maximizing thermal and exergetic performance vary with different third pump outlet pressures. The study also demonstrates that R717 outperforms water in terms of specific net electricity generation, thermal and exergetic performance [48]. In another experimental study, a comparison is made between basic and regenerative ORC systems at different temperature levels using R227ea, R245fa, and R134a as operating fluids. Surprisingly, the simpler basic ORC indicates better efficiency under specific conditions, highlighting the importance of investigating both configurations. Additionally, this study explores the potential use of fourth-generation refrigerants with reduced environmental impact [49]. Another experimental study compares three different ORC systems with six operating fluids under the identical excess heat conditions, utilizing exergetic performance as the goal function. The results indicate that the double-stage regenerative (DRORC) system outperforms the others in terms of thermal and exergetic performance, with R11 and R141b recommended as proper operating fluids for ORCs. R-ORC generally offer better efficiency according to the basic ORC [50]. In another experimental study, a comparison is conducted between an R-ORC and a simple ORC (SORC) using different operating fluids to harness low-temperature excess heat from an industrial source (160–200 °C). Various fluids were analyzed, and R-600a outperformed R-245fa, demonstrating a 3 % higher power output. The R-ORC proved to be more efficient, with an 8–15 % improvement over the SORC, achieving maximum efficiencies of 12 % (R-600a) and 10.7 % (R-1233zd-E) at a 15 °C superheat level. An increase of 10 % in expander efficiency resulted in a thermal efficiency improvement of up to 13.5 %. Additionally, the SORC's thermal efficiency increased by 34–40 % as the evaporation temperature upgraded from 90 °C to 130 °C under fixed conditions [51]. In another experimental study, the thermo-economic optimization of ORC systems for excess thermal energy improvement is discussed. It examines both the simple ORC and R-ORC configurations under constant thermal energy resource conditions employing the NSGA-II. The primary objectives are to improve thermal performance and minimize specific investment costs. The study identifies R245fa as the most proper operating fluid. It is observed that a single-stage R-ORC enhances performance by 1.01 % at an additional cost of $187/kW, while a double-stage R-ORC upgrades performance by 1.45 % with an additional cost of $297/kW. Operating parameters, particularly evaporation pressure, have an important effect impact on both thermal performance and cost [52].

### 4.2 Theoretical studies for ORC and R-ORC

Another theoretical study, a novel R-ORC utilizing a vapor injector as a regenerator shows better thermal performance than the basic ORC under specific conditions, with evaporating and condensing temperatures playing a crucial role for R123 fluid [53]. A theoretical recent study introduces a comprehensive thermo-economic optimization approach for both standard and regenerative ORCs. It examines the effects of varying heat source parameters, explores diverse expander options, and adheres to EU F-gas regulations with consideration for four different working fluids. The optimization primarily focuses on key variables like evaporating pressure, condensation temperature, heater and cooler pinch points, and recuperator effectiveness. The results indicate that Cyclopentane is the top choice for smaller applications, while propane and R1234ze outperform others in larger systems. Expander selection, influenced by operational conditions, significantly impacts ORC's economic performance. The study underscores the heater's pinch point as a critical factor, with the cooler's pinch point being less influential. R-ORCs suit high-temperature closed-loop systems well, but are less suited for open-loop configurations. An economy of scale impact is seen, with specific investment costs ranging from 15,067 €/kWe to 770 €/kWe, showing variation across different system capacities and configurations [54]. Another theoretical study conducts thermal-economic optimization for a parallel dual-pressure evaporation and two-stage R-ORC (DTRORC) utilizing a mixed operating fluid. The research involves a comparison of DTRORC with other ORC systems, focusing on both thermodynamic and economic performance. The results demonstrate that DTRORC achieves a 1.75 % higher thermal performance, 3.5 % higher exergy performance, and 1 kW greater net electricity compared to the dual-stage evaporate ORC (DORC), as well as a 0.8 % higher thermal performance, 2.5 % higher exergy performance, and 0.5 kW greater electricity according to the double-stage R-ORC (DRORC) [55]. Another theoretical study compares single-stage and double-stage R-ORCs SSRORC and DSRORC utilizing medium-grade solar thermal energy with Therminol 55 and R141b as the operating fluid. DSRORC outperforms SSRORC, with 7.9 % higher net electricity generation and thermal performance. Exergy analysis also shows DSRORC with 7.69 % higher exergetic performance [56]. Another theoretical study examines simple and regenerative ORC systems for excess thermal energy improvement with some fluids (R113, R114, R227ea, R245fa and R600a). Various fluid options are examined, and the analysis reveals that the regenerative ORC exhibits higher thermal performance in comparison to the simple ORC, and this improvement is further enhanced with an increase in turbine inlet pressure [57]. Another theoretical study explores the optimization of R-ORC setups, including both open and closed preheater configurations. Various operating fluids (R1234yf, butane, Toluene, Cyclohexane, Cyclopentane, Isohexane, n-pentane) are considered, and their thermal energy performances are compared to a simple ORC. R-ORC system is discovered to be more suitable for dry fluids than standard ORC system, yielding related performance winnings changing from 4.98 % to 9.29 %, while the closed preheater configuration shows minimal performance improvement [58]. Another theoretical study explores the utilization of some fluids in R-ORC to harness low-grade thermal energy resources for power generation. The range of organic working fluids being scrutinized encompasses R113, R245ca, R123, and isobutene, with their boiling points spanning from −12 °C to 48 °C. The research undertaking involves a comprehensive analysis and comparative assessment of the R-ORC against the conventional standard ORC. The primary objective is to pinpoint the configuration that yields the highest thermal efficiency while minimizing irreversibility. Both configurations undergo a thorough evaluation employing a holistic approach, incorporating both the 1st and 2nd Laws of Thermodynamics. This analysis encompasses the manipulation of specific system operating parameters across a spectrum of reference temperatures and pressures to yield a comprehensive perspective on their performance characteristics. The findings indicate that the R-ORC outperforms the standard ORC by achieving greater efficiency and reducing the excess heat required to generate the equal power, all while minimizing irreversibility [59]. Another theoretical work reviews the potential of ORC in mitigating the environmental impact of conventional energy sources like fossil fuels by utilizing industrial waste heat from renewable energies. It discusses various ORC configurations, including recuperative and regenerative cycles, two-stage cycles, and hybrid systems, emphasizing their higher efficiency compared to basic single-stage cycles. While it is evident that hybrid systems demonstrate the highest levels of efficiency, it's important to note that they necessitate exceptionally elevated operating temperatures. In contrast, the majority of ORC facilities typically operate within a power output range spanning from 1 kW to several tens of kW. These systems often employ micro turbines in conjunction with plate heat exchangers to achieve their intended results [60]. Another theoretical paper conducts a thermodynamic and economic evaluation of two solar ORC configurations, namely the two-stage series ORC (TSORC) and the regenerative TSORC (R-TSORC), in the context of a hot, non-temperate climate for a solar project. The study assesses various thermodynamic parameters, including electricity generation, thermal and exergetic efficiency, as well as economic factors like electricity generation cost, payback time, and rate of return on investment. The results indicate that, when supplied with the same thermal potential from the solar system, R-TSORC requires a higher mass flow rate of heat transfer fluid but features a shorter total length of receiver tube compared to TSORC. However, R-TSORC exhibits more competitive performance for all variations when compared to TSORC [61].

## 5 Conclusion

The significant conclusion drawn from the comparison between the ORC and the R-ORC highlights the critical role that these two thermal conversion technologies can potentially play in terms of energy conversion efficiency and sustainable energy production. ORC, utilizing temperature differentials derived from renewable and non-renewable energy sources, possesses a substantial potential for electricity generation, often standing out for its capacity to yield highly efficient energy outputs from low-temperature sources. R-ORC, on the other hand, further mostly enhances these advantages by optimizing the energy conversion process through regenerative heat recovery, thereby increasing system efficiency. When the studies are examined, it is seen that R-ORC performs better than the basic ORC. When the aforementioned studies are examined, it is evident that R-ORC outperforms ORC in terms of both energy and exergy efficiency. Specifically, when looking at energetic efficiency, R-ORC is found to be 1.83 % and 25.5 % more efficient. In terms of exergetic efficiency, it is approximately 7.69 % better. Furthermore, due to these efficiency improvements, R-ORC appears to offer a better economic contribution compared to ORC. These comparative results underscore the potential of conversion technologies like ORC and R-ORC in achieving sustainability goals within the renewable energy field and serve as essential guiding principles for making design and implementation decisions regarding the preferred options for energy production and efficiency under specific conditions. These comparative results showcase the unique advantages of both conversion technologies in terms of energy production and efficiency. Furthermore, they provide a vital roadmap for engineering and economic decision-making regarding the preferred system design or implementation under particular circumstances. Technologies such as ORC and R-ORC promote the more efficient use of renewable energy resources and contribute to the growth of the sustainable energy sector. Hence, the future significance of these two technologies is expected to increase, driven by the growing demand for sustainable energy generation and efforts to combat climate change. This study makes a substantial contribution to optimizing system designs for various applications by providing a comprehensive comparison of ORC and R-ORC systems, which in turn significantly enhances the efficiency and effectiveness of renewable energy solutions. In addition to this comparative analysis, the research highlights best practices and identifies critical areas for improvement, fostering an environment of innovation and sustainability within energy technologies. By emphasizing sustainable energy solutions, this research underscores the essential role that advanced cycles play in promoting a cleaner and more sustainable energy future. However, it is crucial to recognize that, despite the notable advantages in energy conversion efficiency offered by ORC and R-ORC cycles, their practical applicability may be constrained by several factors. These include high initial investment costs, the availability of suitable thermal energy sources, and the necessity for specialized equipment and ongoing maintenance. Therefore, a careful evaluation of these aspects is imperative for successful implementation, ensuring that the potential benefits of these advanced systems can be realized in a manner that meets the demands of a rapidly evolving and increasingly competitive global energy market.

## CRediT authorship contribution statement

**Serdal Damarseckin:** Writing – original draft, Visualization, Investigation. **Sebe Yves Junior Kane:** Writing – original draft, Visualization. **Ayhan Atiz:** Writing – original draft, Visualization, Methodology. **Mehmet Karakilcik:** Writing – review & editing, Supervision, Methodology. **Haci Sogukpinar:** Writing – review & editing, Methodology, Investigation. **Ismail Bozkurt:** Writing – review & editing, Supervision. **Saadin Oyucu:** Visualization, Software, Methodology. **Ahmet Aksoz:** Visualization, Software, Methodology.

## Data availability statement

No data was used for the research described in the article.

## Declaration of competing interest

The authors declare that they have no known competing financial interests or personal relationships that could have appeared to influence the work reported in this paper.
